# The Difficult Airway Trolley: A Narrative Review and Practical Guide

**DOI:** 10.1155/2019/6780254

**Published:** 2019-01-27

**Authors:** Martin F. Bjurström, Mikael Bodelsson, Louise W. Sturesson

**Affiliations:** Lund University, Skåne University Hospital, Department of Clinical Sciences Lund, Anesthesiology and Intensive Care, Lund, Sweden

## Abstract

Death and severe morbidity attributable to anesthesia are commonly associated with failed difficult airway management. When an airway emergency develops, immediate access to difficult airway equipment is critical for implementation of rescue strategies. Previously, national expert consensus guidelines have provided only limited guidance for the design and setup of a difficult airway trolley. The overarching aim of the current work was to create a dedicated difficult airway trolley (for patients>12 years old) for use in anesthesia theatres, intensive care units, and emergency departments. A systematic literature search was performed, using the PubMed, Embase, and Google Scholar search engines. Based on evidence presented in 11 national or international guidelines, and peer-reviewed journals, we present and outline a difficult airway trolley organized to accommodate sequential progression through a four-step difficult airway algorithm. The contents of the top four drawers correspond to specific steps in the airway algorithm (A = intubation, B = oxygenation via a supraglottic airway device, C = facemask ventilation, and D = emergency invasive airway access). Additionally, specialized airway equipment may be included in the fifth drawer of the proposed difficult airway trolley, thus enabling widespread use. A logically designed, guideline-based difficult airway trolley is a vital resource for any clinician involved in airway management and may aid the adherence to difficult airway algorithms during evolving airway emergencies. Future research examining the availability of rescue airway devices in various clinical settings, and simulation studies comparing different types of difficult airway trolleys, are encouraged.

## 1. Introduction

Critical airway incidents are arguably the most severe and feared complications to anesthesia practice. The comprehensive 4th National Audit Project (NAP4) of the Royal College of Anaesthetists and the Difficult Airway Society [1, 2] provides the most current detailed analysis of airway complications. In the United Kingdom, over the course of a year, out of 2.9 million general anesthetics performed, 16 airway-related deaths and 133 major complications were found. Closed claims analyses related to management of the difficult airway confirm the low, but critical, risk for brain damage and death [3, 4]. Importantly, detailed reviews of airway incidents show that most of the catastrophic outcomes could have been avoided, given improved, structured management of these emergencies.

When an unanticipated difficult airway scenario unfolds, it is key to act in a structured and coordinated manner, with no unnecessary delays. Essential equipment for management of the difficult airway must be rapidly accessed, and these tools should be logically organized. In contrast, qualitative analysis of the NAP4 data shows that there were often delays in providing airway equipment, even for basic items such as endotracheal tubes (ETTs), stylets, nasopharyngeal airways, and supraglottic airway devices (SADs) [1, 2]. Once a difficult airway situation evolves, the risk of cognitive overload and stress-induced deterioration of decision-making and situational awareness increases [5]. Hence, the design and setup of a dedicated difficult airway trolley (DAT) should, in addition to containing the adequate equipment, also ideally facilitate adherence to difficult airway algorithms to decrease risk of human factor mistakes. Importantly, to reap the benefits of a well-designed DAT, it is crucial that those who use the DAT are knowledgeable about its organization and have acquired expertise on all included devices through clinical training and simulations. As a dedicated DAT is often present in sites providing general anesthesia, this is not consistently the case according to recent audits and surveys [6, 7]. Importantly, as most airway emergencies develop during the induction phase, incidents can occur throughout the anesthetic process, including extubation [8] and all the way to the postanesthesia care unit. Other high-risk locations for difficult airway scenarios include intensive care units (ICUs) and emergency departments where specific patient and environmental factors increase the complexity and challenges of airway management [9].

Despite several national guidelines on management of the anticipated and unanticipated difficult airway [10–20], with separate guidelines for pediatric [21–23], obstetric [24], and intensive care [9, 25] settings, only limited effort has been directed towards developing specific suggestions regarding the contents of a DAT. Many of the guidelines provide relatively generic advice, for example, that the content of the trolley should be set up in accordance with local regulations, chosen based on favorable evidence, or skills and preferences of the individual anesthesiologist. Some guidelines suggest a “minimum equipment” setup which does not suffice for the individual department or hospital.

Here, based on evidence presented in peer-reviewed journals, the most recent national guidelines, and expert consensus, we outline a difficult airway trolley for patients >12 years old, which can be implemented in any hospital location where general anesthesia or advanced airway management is conducted.

## 2. Methods

Given the purpose of the present work, to develop a difficult airway trolley based on evidence presented in peer-reviewed journals, updated guidelines, and expert consensus, we performed a systematic literature search of the MEDLINE, Embase, and Google Scholar databases. Reference lists of retrieved articles were manually searched to identify further relevant literature. Web pages of national airway management groups and societies were scanned for pertinent content. The search activity was conducted in April 2018. After finalization of the manuscript, the search was repeated on 2018-12-03 to confirm that the reference list was updated and completed. The search was primarily limited to English language, but Swedish language articles were also considered. Restriction of article language to English has previously been shown to have minimal impact on findings [26].

Since our search strategy was designed to identify all pertinent updated national and international guidelines on difficult airway management, the following key search variables were applied individually and in combination: airway, guideline(s), intubation, equipment, and management. For the MEDLINE search, we explored the MeSH terms “Airway Management/standards,” “Airway Management/methods,” and “Practice Guidelines as Topic”; and for the Embase search, we explored the Emtree terms “airway obstruction” and “endotracheal intubation.” Inclusion of specific device terms did not improve the search algorithm.

**Figure 1:**
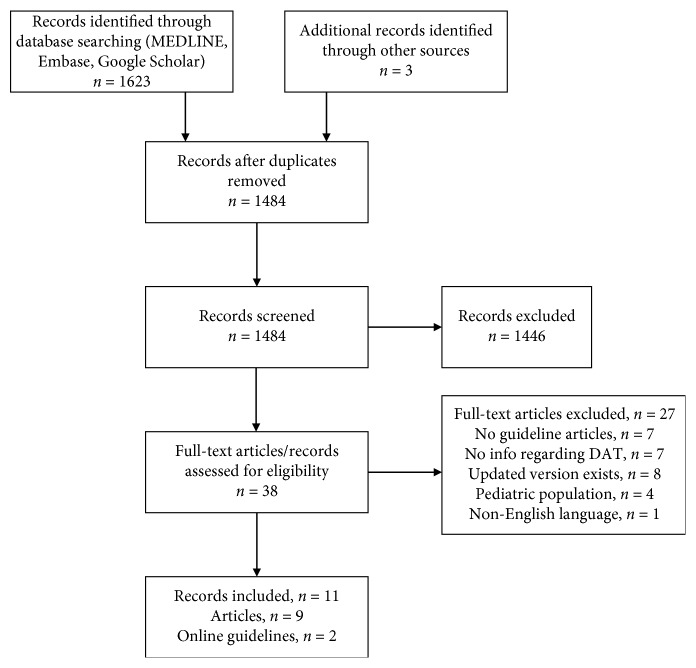
PRISMA flow diagram mapping the guideline article selection process.

For inclusion of a guideline, at least generic advice regarding difficult airway equipment and/or a DAT was required. Articles and guidelines regarding the pediatric difficult airway were excluded since this area was beyond the scope of the review. If an airway society had provided >1 version of a guideline, only the most recent version was considered for inclusion.  Figure 1 shows the PRISMA flow diagram mapping the guideline article selection process. Only one article was excluded based on the language criterion [27].

## 3. Results

### 3.1. Included Guidelines

Through the systematic search process, eleven guidelines with highly varying degrees of information and details regarding difficult airway equipment were identified. A few of the guidelines below provide extensive review of the evidence for different devices, presented in the main articles or background materials published online. Three of the guidelines (Australia/New Zealand, India, and United Kingdom) entail the majority of advice and detail regarding selection of devices for a DAT [13, 17, 28]. The U.S. [12] guidelines provide general suggestions about which equipment to include; the Canadian [16], German [19], Italian [18], Japanese [20], and Swedish [10] guidelines briefly outline minimum or some mandatory equipment. The two national guidelines focused on unanticipated difficult tracheal intubation in obstetric settings add only limited suggestions regarding selection of equipment [24, 29].  Table 1 summarizes key DAT recommendations from the identified most recent guidelines.

### 3.2. Organization of the Difficult Airway Equipment

Organization and stocking of the DAT according to difficult airway algorithms, to potentially improve adherence to a stepwise progression to alternative airway rescue management plans, is explicitly advocated for by two guidelines [13, 17]. Moreover, standardization of equipment is promoted [10, 13, 17, 24]. In addition to a clear recommendation regarding organization of the drawers, the All India Difficult Airway Association (AIDAA) provides a concise list of mandatory and “desirable” equipment, the latter term reflecting the varying economic conditions for Indian healthcare facilities. The Difficult Airway Society (DAS) webpage provides an excellent resource for “setting up a difficult airway trolley” [30]. The provided material is relatively comprehensive, and some specific advice is given about how to set up a DAT, including drawer layout, stocking, and labeling. No other guideline offers specific instructions regarding drawer content or organization within the trolley. It is almost uniformly concluded that difficult airway equipment should be placed at all locations where anesthesia or intensive care is provided. A “grab-bag” with essential equipment to be taken to emergencies or remote anesthetizing locations is also recommended [17, 28].

### 3.3. Availability of the Difficult Airway Equipment

Rapid provision of essential airway equipment is promoted universally. The acceptable timeframe is described as, e.g., “immediately available” [16], “readily at hand” [19], or “within 60 seconds” [28]. Some guidelines subdivide the airway equipment into different categories, such as essential/minimum/mandatory and supplementary/desirable equipment [17, 28], with attached timeframe for the delivery of supplementary equipment (<5 minutes) in one case [28].

### 3.4. The Difficult Airway Trolley: A Proposition

#### 3.4.1. Organization, Design, and Standardization

**Figure 2:**
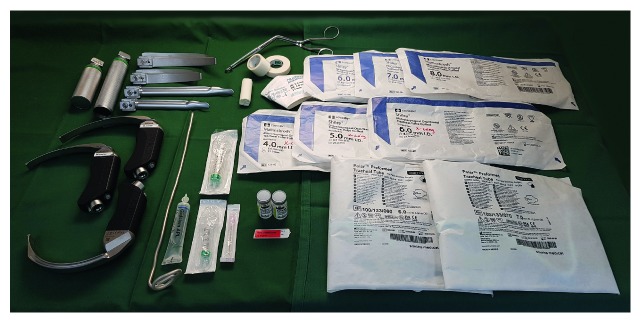
Drawer 1 (plan A) intubation.

**Figure 3 fig3:**
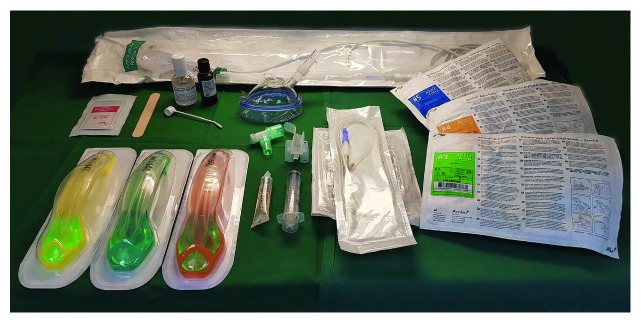
Drawer 2 (plan B) oxygenation via a supraglottic airway device. Note that the videobronchoscope is not included in the drawer but attached to the side of the trolley.

**Figure 4 fig4:**
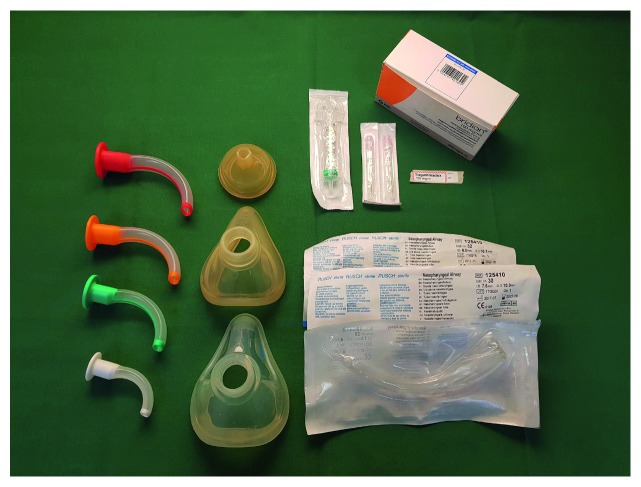
Drawer 3 (plan C) mask ventilation.

**Figure 5:**
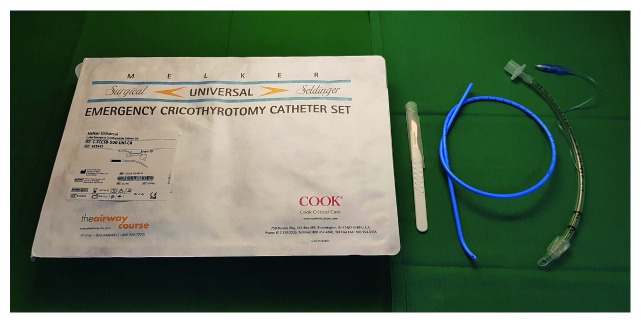
Drawer 4 (plan D) front-of-neck access (FONA). Note that the Frova introducer is not included in the drawer but attached to the side of the trolley.

Over the last decade, the number of devices designed for airway management has increased immensely. Numerous devices fulfill different roles to successfully manage difficult airways. Figures 2 [Fig fig3] [Fig fig4]–5 depict the drawer content of our suggested DAT; Table 2 provides an overview of the contents of the DAT. This DAT does not represent a setup of minimum equipment, but instead all recommended equipment for successful management of most difficult airways. The organization of items is based on the four different plans (A–D) of a widely used difficult airway algorithm (DAS). It remains to be determined whether such an organization improves adherence to guidelines or promotes better clinical outcome. Each drawer corresponds to one step, A–D, in the DAS algorithm. Key concepts which we have implemented in the design of the DAT include logic, simplicity, and standardization. To improve situational awareness and teamwork, we recommend addition of several cognitive aids (see below).

Briefly, we recommend, at least local, standardization of the DAT, so that identical, adequate equipment can rapidly be accessed anywhere in the hospital where general anesthesia or airway management is provided (such as ICUs, emergency departments, operating theatres, and angiography labs). The location of the DAT should be clearly marked. Similarly, the individual drawers must be clearly labelled; different downloadable alternatives can be found online, e.g., on airway organization websites (see below). Through the addition of a fifth drawer, where local additions or adjustments can be made to the DAT (e.g., left-hand laryngoscope blades in the ear, nose, and throat (ENT) anesthesia department or specialized tracheostomy equipment in the ICU), the DAT is ready for use as appropriate. Through this standardized approach, the risk for uncertainty regarding which equipment will be provided is minimized. Training scenarios and simulations using the DAT will ensure that the anesthesiologist is familiar with all included devices. Limiting the number of devices decreases the risk of cognitive overload and delay of actions.

On top of the DAT

(i) Difficult airway algorithm flowchart.
(ii) Brief cognitive aids.
(iii) Contact details (direct access phone numbers) to ENT and senior anesthesiology/intensive care physician resources.
(iv) Stopwatch.
(v) Monitor for use with videolaryngoscope and/or flexible intubating videobronchoscope, depending on brand/type. Alternatively, if the videolaryngoscope equipment and flexible intubating videobronchoscope equipment are separate units with individual monitors, clear, brief directions on top of the DAT to bring videolaryngoscope and video bronchoscope should be provided.

On the side of the DAT

(i) Introducers
(ii) Airway exchange catheter
(iii) Videobronchoscope (regular size)

*Comments*: We strongly recommend clear, brief cognitive aids. For example, the Swedish Society of Anaesthesiology and Intensive Care includes two text boxes in their most recent difficult airway algorithm, which may be printed on a DAT. One box “principles” with the following contents: summon help, maximally three attempts/technique, and flow O_2_; one box “analyze every two minutes”: help summoned? Anesthesia depth, muscle relaxation? Facemask ventilation possible? Possible to wake up patient? Hypoxia (SpO_2_ < 90% and decreasing)? To emphasize the importance of preinduction difficult airway prediction, we also recommend including a cognitive aid, for example, stating “Have you conducted a complete airway evaluation of the patient?” Moreover, we recommend including a difficult airway algorithm flowchart, such as the freely downloadable images provided by the DAS [31]. All cognitive aids and flowcharts should preferentially be laminated.

Regarding videolaryngoscope/videobronchoscope, budget restraints might preclude the mounting of one monitor per DAT unit. In this case, clear instructions must be provided on top of the DAT to bring portable videolaryngoscope and videobronchoscope units to avoid arriving at the scene of a difficult airway scenario without these essential items. If a monitor is attached to the DAT, separate videolaryngoscope blades are placed in drawer 1 (see below), and one regular-size videobronchoscope is attached to the DAT. Intentionally, only one size is provided to avoid confusion and unnecessary delay of action.

Introducers are essential components of a DAT, and emerging data may motivate more widespread use of these low-cost, high-efficacy devices during difficult airway conditions [32]. We recommend including two different types of introducers: one with an angled tip (e.g., Frova introducer) [33] and one thin, soft, and flexible bougie [13]. The stopwatch has been included to improve situational awareness and to increase the likelihood of progressing adequately through the difficult airway algorithm as a scenario evolves. Similarly, to avoid misunderstanding regarding which expert resources to contact immediately, a few clearly marked phone numbers are provided. The Aintree intubation catheter is a valuable piece of equipment, but it is the opinion of the authors that it could be stored elsewhere outside the DAT, to be available within a few minutes.

  Drawers 1–4

Each drawer should be clearly labelled externally to indicate its contents; such signage can be developed by the individual department. Nevertheless, excellent downloadable images are available online, e.g., those created for the Vortex emergency airway cart [34] or the DAS DAT [30]. Preferentially, the utilized images could be preprinted on the drawers when the trolley is ordered to increase durability and facilitate cleaning.

Drawer 1 (plan A) intubation (Figure 2)

(i) Laryngoscope handles: standard and short
(ii) Laryngoscope blades: Macintosh sizes 3 and 4 and Miller sizes 2 and 3
(iii) Videolaryngoscope blades: several different types (if monitor attached to the DAT)
(iv) Endotracheal tubes size 5.0, 6.0, 7.0, and 8.0 and extralong tubes 4.0, 5.0, and 6.0
(v) Nasal endotracheal tubes size 6.0 and 7.0
(vi) Stylet
(vii) Lubrication gel
(viii) Syringe 10 ml (for cuff inflation)
(ix) Magill forceps
(x) Cognitive aid indicating importance of continuous waveform capnography
(xi) Adhesive tape, wide, and narrow
(xii) Bite block
(xiii) Syringe 5 ml (for rocuronium)
(xiv) Aspiration cannula
(xv) Rocuronium 10 mg/ml
(xvi) Preprinted labels “Rocuronium”

*Comments regarding drawer 1*: Availability of laryngoscope blades of alternate design (e.g., straight blade (Miller) or blade with adjustable hinged tip (McCoy)) and size is recommended by several guidelines [12, 13, 19, 28, 29]. Endotracheal tubes of varying sizes [10, 12, 17, 18, 28], specific sizes/lengths [18, 28], material (armored) [18, 28], stylets, guides, tracheal introducers, and airway exchange catheters [10, 12, 13, 17–20, 28] are considered essential. The DAS specifically recommends including the Aintree intubation catheter (Cook Medical, Bloomington, USA) [13]; the Aintree intubation catheter is denominated as desirable by the AIDAA [17]. The Magill forceps is commonly recommended [12, 17–19]. Failure to monitor exhaled carbon dioxide accounted for a disproportionate number of deleterious airway incidents in the NAP4 report, particularly in the emergency department and ICU; hence, it is not surprising that several guidelines promote the inclusion of a capnograph [12, 13, 17, 28]. Indeed, in the DAS guidelines, the pivotal role of capnography to detect esophageal intubation or accidental extubation is emphasized: “A continuous capnography waveform with appropriate inspired and end-tidal values of CO_2_ is the gold standard for confirming ventilation of the lungs. Capnography should be available in every location where a patient may require anaesthesia” [13].

The advent of commercially available videolaryngoscopes in 2001 can be considered a paradigm shift for the management of unanticipated and anticipated difficult airways. Although there are inherent limitations associated with videolaryngoscopy [35], these devices have overall been shown to improve laryngeal view and success rate of tracheal intubation [36]. Today, videolaryngoscopy constitutes the first employed backup technique after failed intubation attempts using direct laryngoscopy [37]. Most guidelines emphasize this pivotal role of the videolaryngoscope and its natural place in the difficult airway armamentarium [10, 12, 13, 16, 19, 20, 24, 29]. However, the Canadian Airway Focus Group lists the videolaryngoscope as only one of several options when direct laryngoscopy has failed and does not specifically stress the importance of an immediately available videolaryngoscope, emphasis is rather placed on the individual practitioner's judgement [16]. Surprisingly, the Australian and New Zealand College of Anaesthetists (ANZCA) does not include a videolaryngoscope in its essential equipment list; instead, it is listed among supplementary equipment, to be available within 5 minutes [28]. When the Italian guidelines were conceived, videolaryngoscopy was not yet used in a widespread manner; hence, these devices were not considered mandatory [18]. Similar to the case of the Aintree intubation catheter, AIDAA lists videolaryngoscopes as desirable [17]. The most recently released guideline, written by the Swedish Society of Anaesthesiology and Intensive Care, goes as far as recommending the use of a videolaryngoscope as the first-line intubation device for rapid sequence induction intubation, particularly in obstetric settings [10]. There is limited recommendation regarding specific choice of videolaryngoscope; future comparative studies are encouraged [36, 38].

Regarding the endotracheal tubes, it is very important to mark the extralong variants clearly to avoid confusion when these are needed. Also, note that the soft precurved nasal tubes can be used with a flexible intubating videobronchoscope. The precurved nasal tubes are longer than the extralong ETTs; hence, particularly in tall patients, these tubes better accommodate safe nasal intubation with lower risk of tube displacement. Whereas the McCoy laryngoscope has been shown to perform well in various scenarios [38], it is the opinion of the authors that there is limited use for this type of laryngoscope blade, particularly in the era of videolaryngoscopy. Straight laryngoscope blades (Miller) have been included to facilitate a paraglossal technique which may generate a better view of the laryngeal inlet, for example, in cases of macroglossia or in patients with a long distance between the teeth and glottis such as acromegalia [39]. If videolaryngoscope blades are placed in this drawer, several different blades should be available, including a hyperangulated type, such as the C-MAC D-blade or the GlideScope LoPro blades. As compared to conventional video Macintosh blades, the hyperangulated blade is more curved to improve glottic exposure and may be advantageous to use under difficult intubation conditions [40]. The hyperangulated shape typically requires the use of a correspondingly curved stylet, to guide the ETT to the laryngeal inlet. Adequate muscle relaxation is a necessity in the difficult airway scenario. Short-acting suxamethonium effect will likely be subsiding by the time the DAT is brought forward. Rocuronium has been included in the drawer, to ensure neuromuscular blockade during attempts to secure the airway.

Although transnasal humidified rapid-insufflation ventilatory exchange (THRIVE) is receiving increasing attention in the setting of difficult airway management, only one organization proposes inclusion of such a device at this stage [17]. Two obstetric guidelines recommend high-flow oxygen administration (5–15 L/min) through a nasal cannula during intubation attempts [24, 29].

Drawer 2 (plan B) oxygenation via a supraglottic airway device (Figure 3)

(i) Two different types of 2nd generation SADs. Sizes 3, 4, and 5 of each of these two SADs
(ii) Lubrication gel
(iii) Syringe 20 ml (for cuff inflation)
(iv) Orogastric tube sizes 12 and 14
(v) Adjuvants for flexible videobronchoscopic-guided intubation, e.g., endoscopy mask, breakaway oropharyngeal airway, swivel connector, spray solution lidocaine 40 mg/ml and 100 mg/ml, antifog solution, and tongue depressors

*Comments regarding drawer 2*: Plan B entails the placement of a SAD to oxygenate the patient. Importantly, many factors which may underlie failed intubation (plan A) do not directly affect the insertion of a SAD [41]. Supraglottic airway devices are consistently recommended to be readily available for rescue ventilation [10, 12, 13, 15, 17–20, 28, 29]. Five of the guidelines specifically recommend abandoning 1st generation SADs (such as the classic LMA, cLMA) and include only 2nd generation devices [9, 13, 17, 24, 29]. In a recent editorial, Cook and Kelly stated that “the cLMA was devised more than 30 years ago, and the prefix “classic” might now indicate that it is a “vintage” device rather than a “state-of-the-art” one” [42]. The rational for this shift is that 2nd generation devices reduce the risk of aspiration through a drainage port which facilitates insertion of a gastric tube for drainage of the stomach, and 2nd generation devices have higher sealing pressures, which decreases leakage during positive pressure ventilation. Additionally, 2nd generation devices serve as better conduits for flexible videobronchoscopic intubation as compared to the 1st generation SADs. However, among the SADs, the cLMA has by far accumulated the largest body of evidence for use in difficult airway situations (>300 publications) [43]. Nevertheless, the advantages of 2nd generation devices are apparent and increasing evidence supports the use of these devices for rescue ventilation. There are few comparative studies examining the most widely used 2nd generation SADs [44–47]. Overall, the three 2nd generation devices with the, to date, strongest evidence from large longitudinal studies and meta-analyses are the i-gel (Intersurgical, Wokingham, UK), the LMA Supreme (SLMA; Teleflex Medical Europe Ltd, Athlone, Ireland), and the Proseal LMA (PLMA; Teleflex Medical Ltd). Cook and Kelly compared the most commonly used SADs using a simple scoring system taking into account seven factors: overall insertion success, speed of insertion, quality of ventilation, airway seal, aspiration protection, avoiding airway trauma, and avoiding sore throat [42]. Out of a maximum score of 22, the three top devices were the i-gel (19), PLMA (18), and SLMA (17) (e.g., compared to cLMA (14) and the intubating LMA, ILMA (15)). Another aspect related to the SAD as an essential component of the DAT is the inclusion of SADs of different sizes, to optimize placement in the individual patient [13, 19, 28]. In older versions of guidelines, and the updated ANZCA guidelines, specific recommendation is made for use of the ILMA [28]. Although there is a substantial body of evidence for successful blind intubation via intubating SADs [43], in our opinion, flexible videobronchoscopic-aided intubation through newer SADs precludes the use of blind techniques.

Flexible fiberoptic endoscopes, or today more commonly used flexible intubating videobronchoscopes are increasingly considered to be essential components of the DAT [10, 12, 13, 19]. These devices enable rescue intubation through a successfully placed SAD, although the technique may be challenging related to different types of SADs. For example, it has been reported that videobronchoscope-guided intubation through the SLMA may be more challenging compared to the i-gel or PLMA [48]. ANZCA does not include flexible videobronchoscopes on its essential equipment list but states that the device should “ideally be available within 5 minutes” [28]. According to the Italian guidelines, these devices should be available upon request (no timeframe specified) [18], and the AIDAA lists the flexible videobronchoscopes as desirable equipment [17]. DAS recommends the inclusion of a flexible videobronchoscope on top of the DAT and specifies a number of adjuvants to facilitate bronchoscope-guided intubation [30].

Drawer 3 (plan C) mask ventilation (Figure 4)

(i) Facemask sizes 3 and 4
(ii) Neonatal facemask size 0
(iii) Oropharyngeal airway different sizes, e.g., 7, 9, 10, and 11 cm
(iv) Nasopharyngeal airway sizes 6.0, 7.0, and 8.0
(v) Sugammadex 100 mg/ml, 2 ml/vial, 8 vials
(vi) Syringe 10 ml
(vii) Aspiration cannula
(viii) Preprinted labels “Sugammadex”

*Comments regarding drawer 3*: Basic equipment for maintenance of airway patency and ventilation, such as facemasks and oropharyngeal and nasopharyngeal airways of different sizes, are commonly mandated [10, 13, 17, 19, 28, 29]. The neonatal mask facilitates ventilation over a tracheostomy stoma. In case of successful mask ventilation, waking up the patient might during plan C be an option. For this situation, sugammadex has been included, to enable rapid reversal of neuromuscular blockade (NMB) with rocuronium (or vecuronium) [49, 50]. The included total of 1600 mg sugammadex is sufficient to immediately reverse NMB in a 100 kg patient (16 mg/kg).

Drawer 4 (plan D) emergency invasive airway access (Figure 5)

(i) Emergency cricothyrotomy catheter set
(ii) Endotracheal tube size 6.0
(iii) Scalpel blade 10

*Comments regarding drawer 4*: When a “can't intubate, can't oxygenate” (CICO) situation has been declared, immediate preparations for front-of-neck access (FONA) must be prompted. Equipment for FONA is universally recommended as a last resort to manage the difficult airway [9, 10, 12, 13, 16, 17, 19, 20, 24, 28, 29]. The DAS guidelines [9, 13, 24] specifically recommend a surgical technique, whereas the Italian guidelines advocate the Seldinger technique [18]. According to the NAP4 report, released several years after the publication of the Italian guidelines, the use of scalpel cricothyroidotomy had the highest success rate compared to other FONA techniques. The DAS guidelines include a detailed stepwise guide for execution of the surgical technique, both in case of a palpable cricothyroid membrane (“stab, twist, bougie, tube”) or an impalpable membrane [13]. Onrubia et al. have recently provided an excellent review of different FONA techniques [51].

We recommend the surgical technique which only necessitates provision of three items: scalpel (blade 10), bougie (e.g., a Frova introducer), and a tube (6.0). As the Frova introducer passes easily through a size 6.0 tube, it is noteworthy that also a size 5.0 tube would work, with the advantage of lower resistance passing the tube through the skin. Given that many anesthesiologists are proficient in placement of central venous lines and percutaneous tracheostomies, the Seldinger technique kit for surgical cricothyrotomy may be an alternative [13]. Combination sets are available, which in addition to these items include equipment for the Seldinger technique (e.g., MELKER Universal Cuffed Emergency Cricothyrotomy Catheter Set). For the event that sugammadex has been administered during execution of plan C, another NMB agent than rocuronium or vecuronium may be required to ensure optimal conditions for establishing a surgical airway. Atracurium can be considered; however, its potential histamine-releasing effects may have negative hemodynamic implications. In this scenario, due to high failure rates and complications associated with cannula cricothyroidotomy [4] and to limit the number of options, devices to facilitate cannula techniques have been omitted in this drawer. A small set for sterile skin preparation may be included in this drawer, but under the circumstances of emergency FONA, it is not considered mandatory.

  Drawer 5 (optional, customized equipment)

This drawer enables addition of further specialized equipment, pertinent to specific areas of the hospital, e.g., ENT-operating rooms and ICUs. Examples include, but are not limited to, left-hand laryngoscope blades, Combitubes, and equipment for management of tracheostomies. The Ventrain device (Ventinova, NL) may be considered for inclusion in this drawer, although there is to date only limited literature supporting its efficacy [52, 53].

## 4. Discussion

Despite decades of research, prediction of a difficult airway remains challenging. Indeed, no bedside test designed to predict a difficult airway has shown both high sensitivity and specificity [54, 55]. Nevertheless, careful patient assessment and airway evaluation are crucial for identification of many potentially difficult airway situations prior to induction of anesthesia [56]. The importance of preparedness, team briefing, and development of strategies before airway instrumentation must be underscored, and an optimally designed and well-stocked DAT is no guarantee for successful airway rescue if there is a lack of strategy and nontechnical skills [57]. Once a difficult airway is encountered, swift and structured management is key to prevent detrimental outcomes.

The purpose of this review was to develop a difficult airway trolley based on current national and international difficult airway guidelines. Although most guidelines conclude that difficult airway equipment must be easily accessible and logically organized, only limited effort has previously been made to present concrete solutions for a DAT. Whereas there is to date a lack of evidence regarding comparative performance of airway devices, it is our firm belief that the provided DAT can serve as a useful tool for any hospital seeking to optimize their portable difficult airway equipment unit.

Our proposed DAT is organized according to one of the most widely implemented difficult airway algorithms [13], with each of the top four drawers representing one management plan. Such design may facilitate logical stepwise progression through the algorithm as a difficult airway scenario evolves. Although we have attempted to optimize the design of our DAT, there is always potential for improvement through modification of details. For example, whereas we believe that placement of rocuronium, sugammadex, and atracurium in different drawers may facilitate execution of, and adherence to, the plan A–D strategies, there might during high-stress conditions arise confusion regarding requisition of these drugs. Moreover, recently, experts have argued for merging of plans B and C, to better reflect “a phase of airway rescue, attempting SGA placement interspersed with attempted facemask ventilation” [9]. Despite this argument, we believe that a subdivision of the plans and the corresponding drawers serves a conceptual purpose, and additionally limits the amount of equipment in each drawer, reducing the risk for decision delay. According to our own experience, there are many examples of DATs which more resemble an accumulation of airway devices over the years than a logically designed unit. This unfortunate phenomenon may be due to poorly thought through design plans, but also disagreement regarding which airway management sequence or algorithm to implement. Concepts and elements which we have strived to include in our DAT are logic and simplicity, both regarding selection of equipment and decision-making. Cognitive overload is a major human factor at play during difficult airway situations; we hope that our “less-is-more” layout can reduce this risk [58].

Although the DAS has provided a rare and high-quality online guide for the construction of a DAT [30], we think our difficult airway equipment unit may hold a few advantages. For example, somewhat confusing, LMAs are included in both drawer 2 and drawer 3 of the DAS trolley. Although the most recent guidelines concerning critically ill adults firmly recommend inclusion of only 2nd generation SADs [9], the referenced DAT leaves open the option for a 1st generation device, and also includes the ILMA [30]. This amount of devices may in our opinion be too high for decisive management. Indeed, ANZCA states: “it is potentially hazardous to overstock containers of this type” [28]. Also, we believe that some of the devices for cannula cricothyroidotomy included in drawer 5 could be stored elsewhere. For a complete appreciation of the differences between our proposed difficult airway equipment unit and the above-referenced DAT, the interested reader is encouraged to make a detailed comparison.

There are several limitations to the present work. Most importantly, due to the infrequency and nature of unanticipated difficult airway events, clinical prospective randomized studies examining airway equipment and DATs are difficult to perform. Simulations and manikin studies provide some evidence, but the extrapolation of results from such studies may have limited value for real-life difficult airway scenarios. Analyses of airway incidents have highlighted several problems related to management of difficult airways, but there is overall a lack of head-to-head studies comparing different airway devices under clinical difficult airway conditions. Two of the difficult airway management guidelines (Australia/New Zealand and United Kingdom) have already provided relatively extensive evaluation of the evidence underlying use of several essential airway devices. Hence, we omitted replication of these comprehensive device reviews, to increase focus on the creation of an “optimal” DAT. Naturally, there is currently no high-level evidence to support that the presented DAT is superior to previous attempts. To our knowledge, no studies have compared different types of DATs in clinical or simulation settings. We encourage such studies to evaluate whether DATs organized according to difficult airway algorithms, such as ours, the DAS DAT [30], or the Vortex Emergency Airway Cart [34], convey significant benefits over more traditional DATs. Furthermore, the organization and contents of our DAT might be enhanced through feedback from users. Globally, there is to date a paucity of knowledge regarding availability, organization, and stocking of portable difficult airway equipment units in hospitals. To remedy this knowledge gap, we are currently conducting a nationwide study examining the conditions in Sweden.

## 5. Conclusions

To conclude, here, we provide a pragmatic and practical guide for the design and organization of a difficult airway trolley for patients >12 years old. Although the NAP4 report has already increased awareness and led to improved provision of difficult airway equipment overall [59], we believe that our work can contribute to highlight this area. Due to constant evolution of new airway equipment and modifications to established devices, the recommended content of our DAT is naturally subject to revision. We recommend standardization and conformity of difficult airway equipment throughout locations in the hospital where airway management and anesthesia are administered. To maximize the benefits of a DAT, it is essential that those who use the DAT are familiar with its organization and are proficient with all available tools. Improved accessibility and contents of a difficult airway trolley can potentially reduce morbidity and mortality relating to difficult airway management.

## Figures and Tables

**Table 1:** National and international guidelines: general recommendations regarding difficult airway equipment.

Nations, year	Reference	Publication type	Recommendations regarding difficult airway equipment
Australia/New Zealand, 2012	[11, 28]	Online (ANZCA web page)	List of essential (minimum) equipment. Additional items “may be added at the discretion of each individual department.” List of supplementary equipment. Emphasizes careful selection of items.

Canada, 2013	[15, 16]	Peer-reviewed article	No specific recommendations regarding content of a DAT. General advice regarding immediate availability of equipment for difficult airway management. Emphasis on the need for adequate equipment in obstetrical units.

Germany, 2015	[19]	Peer-reviewed article	List of minimum equipment for the anesthesiologists' workstation. Airway management equipment should be readily available also in the PACU and ICU. No comments regarding a DAT.

India, 2016	[17]	Peer-reviewed article	Specific suggestions for content of a difficult airway cart, including drawer organization. List of mandatory equipment and desirable equipment.

India, 2016	[29]	Peer-reviewed article	Guidelines focused on unanticipated difficult tracheal intubation in obstetric patients. Brief recommendations regarding selection of equipment.

Italy, 2005	[18]	Peer-reviewed article	List of mandatory devices and devices which should be available upon request.

Japan, 2014	[20]	Peer-reviewed article	Rescue airway devices should be accessible “within seconds from any operating room,” e.g., in a DAT. Limited recommendations regarding specific equipment.

Sweden, 2018	[10]	Online (SFAI web page)	DATs with standardized equipment at all sites where anesthesia or intensive care is provided. Brief, general advice regarding equipment.

United Kingdom, 2015	[13, 30]	Peer-reviewed article, online (DAS web page)	Detailed recommendations regarding setup, organization, and implementation of a DAT. Advice about the design and contents of an ideal DAT. Emphasizes limiting the number of devices to improve decision-making.

United Kingdom, 2015	[24]	Peer-reviewed article	Guidelines concerning difficult/failed tracheal intubation in obstetrics. Standardization of airway equipment within the hospital is recommended. Limited recommendations regarding which equipment to include in a DAT.

ANZCA = Australian and New Zealand College of Anaesthetists; DAS = difficult airway society; DAT  =  difficult airway trolley; ICU = intensive care unit; PACU = postanesthesia care unit; SFAI = Svensk förening för anestesi och intensivvård (Swedish Society of Anaesthesiology and Intensive Care).

**Table 2:** An overview of the organization and contents of the DAT.

Part of the DAT	Contents	
On top	Difficult airway algorithm flowchart Cognitive aids Direct access phone numbers to ENT and anesthesiology/intensive care physician resources Stopwatch	Monitor(s) for videolaryngoscope/videobronchoscope alt. Clear directions to bring videolaryngoscope and videobronchoscope

On the side	Introducers Airway exchange catheter Videobronchoscope (regular size)	

Drawer 1	Laryngoscope handles: standard, short Laryngoscope blades: Macintosh 3 and 4; Miller 2 and 3 Videolaryngoscope blades (if monitor attached to the DAT) Endotracheal tubes 5.0, 6.0, 7.0, and 8.0 Extralong endotracheal tubes 4.0, 5.0, and 6.0 Nasal endotracheal tubes 6.0 and 7.0 Stylet Lubrication gel	Syringe 5, 10 ml Magill forceps Adhesive tape, wide and narrow Bite block Aspiration cannula Rocuronium 10 mg/ml Preprinted labels “Rocuronium” Cognitive aid (continuous waveform capnography)

Drawer 2	Two different types of 2nd generation SADs size 3, 4, and 5 Lubrication gel Syringe 20 ml Orogastric tube size 12 and 14	Adjuvants for flexible videobronchoscopic-guided intubation: endoscopy mask, breakaway oropharyngeal airway, swivel connector, spray solution lidocaine 40 mg/ml and 100 mg/ml, antifog solution, and tongue depressor

Drawer 3	Facemask size 3 and 4 Neonatal facemask size 0 Oropharyngeal airways (e.g., 7, 9, 10, and 11 cm) Nasopharyngeal airway 6.0, 7.0, and 8.0	Sugammadex 100 mg/ml, 2 ml/vial, 8 vials Syringe 10 ml Aspiration cannula Preprinted labels “sugammadex”

Drawer 4	Emergency cricothyrotomy catheter set Endotracheal tube 6.0	Scalpel blade 10 Frova introducer

Drawer 5	Optional, customized equipment (e.g., left-hand laryngoscope blades, equipment for management of tracheostomies, and Ventrain device)	

DAT  =  difficult airway trolley; ENT = ear, nose, and throat; SAD = supraglottic airway device.
